# Childhood cancer and ethnic group in Britain: a United Kingdom children's Cancer Study Group (UKCCSG) study.

**DOI:** 10.1038/bjc.1991.347

**Published:** 1991-09

**Authors:** C. A. Stiller, P. A. McKinney, K. J. Bunch, C. C. Bailey, I. J. Lewis

**Affiliations:** University of Oxford, Department of Paediatrics, UK.

## Abstract

We present here the results of the largest study of childhood cancer and ethnic group in Britain, based on 7,658 children treated at paediatric oncology centres throughout the country. Incidence rates could not be calculated and so relative frequencies were analysed by the log-linear modelling method of Kaldor et al. (1990) with allowance made for regional variations in the ages and diagnostic groups of the children included in the study. Children of Asian (Indian sub-continent) and West Indian ethnic origin had similar patterns of incidence for acute lymphoblastic leukaemia to White Caucasians. There was a significant excess of Hodgkin's disease among Asian children compared with Caucasians with an estimated relative risk (RR) of 2.09; this excess was greatest in the 0-4 age group (RR = 6.67). There were significant deficits of Wilms' tumour and rhabdomyosarcoma among Asian children, each with a frequency around half that among Caucasians, whereas West Indians had a significant excess of Wilms' tumour (RR = 2.55). Asian and West Indian children each had a non-significant twofold RR for unilateral retinoblastoma. The results suggest that the incidence of childhood acute lymphoblastic leukaemia is associated with environmental determinants in the country of residence which are most likely to relate to lifestyle factors. The occurrence of retinoblastoma, Wilms' tumour and Hodgkin's disease in early childhood is apparently related more to ethnicity than to geographical location and may reflect genetic factors or environmental exposures specific to the lifestyle of particular ethnic groups.


Br. J. Cancer (1991), 64, 543-548 © Macmillan Press Ltd., 1991

### Childhood cancer and ethnic group in Britain: a United Kingdom Children's Cancer Study Group (UKCCSG) Study

C.A. Stiller[1], P.A. McKinney[2,4], K.J. Bunch[1], C.C. Bailey[3] & I.J. Lewis[3]

[1]*University of Oxford, Department of Paediatrics, Childhood Cancer Research Group, 57 Woodstock Road, Oxford OX2 6HJ;* [2]*Leukaemia Research Fund Centre for Clinical Epidemiology, University of Leeds;* [3]*Seacroft Hospital, Oncology Unit, Leeds LS14 6UH, UK.*

**Summary** We present here the results of the largest study of childhood cancer and ethnic group in Britain, based on 7,658 children treated at paediatric oncology centres throughout the country. Incidence rates could not be calculated and so relative frequencies were analysed by the log-linear modelling method of Kaldor *et al.* (1990) with allowance made for regional variations in the ages and diagnostic groups of the children included in the study. Children of Asian (Indian sub-continent) and West Indian ethnic origin had similar patterns of incidence for acute lymphoblastic leukaemia to White Caucasians. There was a significant excess of Hodgkin's disease among Asian children compared with Caucasians with an estimated relative risk (RR) of 2.09; this excess was greatest in the 0–4 age group (RR = 6.67). There were significant deficits of Wilms' tumour and rhabdomyosarcoma among Asian children, each with a frequency around half that among Caucasians, whereas West Indians had a significant excess of Wilms' tumour (RR = 2.55). Asian and West Indian children each had a non-significant twofold RR for unilateral retinoblastoma.

The results suggest that the incidence of childhood acute lymphoblastic leukaemia is associated with environmental determinants in the country of residence which are most likely to relate to lifestyle factors. The occurrence of retinoblastoma, Wilms' tumour and Hodgkin's disease in early childhood is apparently related more to ethnicity than to geographical location and may reflect genetic factors or environmental exposures specific to the lifestyle of particular ethnic groups.

Studies of variations in cancer incidence between ethnic groups in the same country can yield important clues in the search for aetiological factors. Changes in incidence among migrant populations may suggest environmental determinants in the host country whereas stable incidence may indicate relatively greater importance of genetic factors. Patterns of childhood cancer incidence in different ethnic groups in the United States are well documented (Kramer *et al.*, 1983; Young *et al.*, 1986; Parkin *et al.*, 1988*a*; Goodman *et al.*, 1989*a*; Goodman *et al.*, 1989*b*) and incidence rates by ethnic group have been published for several other countries, mostly in Asia (Parkin *et al.*, 1988*a*). An increasing proportion of the British childhood population are members of diverse ethnic groups but hitherto little published information on their patterns of cancer incidence has been available. In particular there have been no large scale studies of incidence among children of Indian sub-continent ethnic origin living in Western countries.

The present study was prompted by clinical observations in Leeds of an unusual distribution of cancer among Asian children of which the most striking feature was a high relative frequency of Hodgkin's disease early in life. The register of the United Kingdom Children's Cancer Study Group (UKCCSG) now includes over two thirds of all childhood cancers in Britain, and ethnic group has been recorded for the great majority of patients since 1981. These data have been analysed in order to build up for the first time a general picture of ethnic variations in the occurrence of childhood cancer in Britain.

### Patients and methods

Since the formation of the UKCCSG in 1977, all children with malignant disease who were patients at specialist paediatric oncology centres have been included in the UKCCSG register. The information recorded includes the age and sex of the children and details of the diagnosis. Since 1981 information on ethnic group has also been collected. The present analysis of UKCCSG data is based on 7,658 children in the register who were domiciled in Britain and diagnosed during 1981 onwards. The series represents an estimated two thirds of all cases of childhood cancer diagnosed during this period.

Childhood neoplasms are more appropriately classified by histology rather than primary site. A classification scheme was developed on the basis of histological type by Birch and Marsden (1987) and used for the recent monograph on international childhood cancer incidence (Parkin *et al.*, 1988*a*). We have used a slightly modified version of this scheme for all of the analyses presented here. In this version, the principal changes are that megakaryocytic leukaemia has been included with acute non-lymphocytic leukaemia (ANLL), rhabdoid renal tumour and bone-mestastasising renal tumour of childhood have both been included with Wilms' tumour, and peripheral neuroectodermal tumours have been classified with Ewing's sarcoma if in bone and with soft-tissue sarcoma other than rhabdomyosarcoma and fibrosarcoma if in other sites. Langerhans cell histiocytosis has been excluded since it is not now regarded as a neoplasm.

The register is not population-based and population data are not available by ethnic group, hence incidence rates could not be calculated and the analyses are based on relative frequencies of different diagnostic groups.

The distribution of ethnic groups in Britain varies geographically, and the age distribution of the child population may differ between ethnic groups. The proportion of children referred to paediatric oncology centres, and hence included in the register, varies between regions and diagnostic groups, and older children are generally less likely to be referred (Stiller, 1988). To allow for these confounding factors, the data were analysed by the log-linear modelling method of Kaldor *et al.* (1990) using the GLIM statistical package (Payne, 1987).

As the data were not population-based and incidence rates could not be calculated, a case-control approach was adopted for analysing relative frequencies of each diagnostic group between the ethnic groups (Breslow & Day, 1987). For the

Correspondence: C.A. Stiller.
[4]Present address: Information and Statistics Division, Scottish Health Service Common Services Agency, Trinity Park House, South Trinity Road, Edinburgh EH5 3SQ, UK.
Received 8 March 1991; and in revised form 26 April 1991.

analysis of each successive diagnostic group, all other cancers were used as controls. In the standard GLIM program notation, the model fitted to the data was:

Centre + Age + Sex + Ethnic group . Age.

A further term, Age . Sex, was fitted if there was a significant interaction between these variables. In this notation the plus signs link variables whose effects on the relative risk are additive on the logarithmic scale, i.e. multiplicative on the arithmetic scale. A full point indicates that interactions between the two variables are also fitted.

The variables were all categorical, defined as follows:

| | |
|---|---|
| Centre | One value for each of the 20 centres to allow for regional differences in referral to these centres. |
| Age | Three five-year groups, 0–4, 5–9 and 10–14. |
| Sex | Male and female. |
| Ethnic group | White Caucasian; Asian (of Indian, Pakistani or Bangladeshi ethnic origin); West Indian (Afro-Caribbean); Other and mixed ethnic groups. |

Age was not included in the model for retinoblastoma since 94% of patients were aged 0–4, 6% were aged 5–9 and there were none aged 10–14. Centre was also not allowed for in relation to retinoblastoma as 163 (75%) of all children were registered from one hospital which acts as a national referral centre for this tumour. Laterality was recorded for all but one case of retinoblastoma and the model was also fitted separately for unilateral and bilateral tumours.

### Results

The register contained 7,658 children resident in the United Kingdom with cancer diagnosed during 1981 onwards. Of these, 6,783 (89%) were described as White Caucasian, 366 (4.8%) as Asian, 63 (0.8%) as West Indian and 173 (2.3%) as of other or mixed ethnic group. This last category included a wide variety of ethnic origins, of which the most numerous were mixed Caucasian-West Indian (34 registrations) and Chinese (19 registrations). The ethnic group was not recorded for the remaining 3.6% of registrations. Table I shows the distribution by ethnic group of UKCCSG registrations for the principal types of childhood cancer during 1981–89. Children with brain and spinal tumours are known to be under represented in the register, as are children aged 10–14 in all diagnostic groups (Stiller, 1988). Table II shows the numbers of cases registered among Caucasians and Asians by five-year age group for selected diagnostic groups.

The commonest childhood cancer, acute lymphoblastic leukaemia (ALL) had a similar relative frequency, around 30% of registrations, in all the ethnic groups (Table I). The relative frequency of ALL in each age-group was also similar for Caucasians and Asians (Table II). The immunophenotype of ALL was recorded in 72% of cases and the results for the three main ethnic groups are shown in Table III. There was no evidence of variation in phenotype with ethnic group; in particular all groups showed a similar preponderance of common ALL, especially at age 1–9.

The relative frequency of Hodgkin's disease in Asians was 1.8 times that in Caucasians (Table I). This excess was most marked in the youngest age group and remained substantial among children aged 5–9 but was virtually absent in those aged 10–14 (Table II). West Indian children had a high relative frequency of Hodgkin's disease but this was based on only five cases. The histological subtype was recorded for 98% of cases. Subtypes for Caucasian and Asian children are shown in Table IV. The mixed cellularity subtype accounted for half of the Asian children and nodular sclerosis for around a third, whereas in Caucasians only a quarter of cases were mixed cellularity and half were nodular sclerosis. Of the five West Indian children (all girls), three had nodular sclerosis and one each lymphocyte predominant and mixed cellularity.

Retinoblastoma had a raised relative frequency among Asian children (Table I). The distribution of laterality was markedly different between Caucasians, of whom 101 (52%) had unilateral and 94 (48%) had bilateral tumours, and Asians, whose tumours were unilateral in ten (77%) cases and bilateral in only three (23%). Overall the proportion of bilateral tumours (46%) was higher than the 40% in the most recent British population-based series (Sanders, personal communication). This reflects preferential referral of children with bilateral tumours to specialist centres (Sanders *et al.*, 1988). Among the children with unilateral tumours, nine (9%) of the Caucasians and one (10%) of the Asians had a family history of retinoblastoma, implying that they, in addition to the bilateral cases, had the heritable form of the disease.

Wilms' tumour had a low relative frequency in Asian children and a high relative frequency in West Indians (Table I). The deficit of Wilms' tumour among Asians was spread equally between those aged 0–4 and 5–9 years (Table II). The excess among West Indians was especially striking for

**Table I** Numbers of registrations for childhood cancer in UKCCSG Register by diagnostic group and ethnic group, 1981 onwards

| | | | *Ethnic group* | | | |
|---|---|---|---|---|---|---|
| *Diagnostic group* | *Caucasian* | *Asian* | *West Indian* | *Other and mixed* | *Unknown* | *Total* |
| ALL | 2,113 | 121 | 17 | 53 | 80 | 2,384 |
| ANLL | 410 | 21 | 2 | 11 | 17 | 461 |
| All other leukaemia | 80 | 5 | 1 | 2 | 6 | 94 |
| Hodgkin's disease | 297 | 29 | 5 | 5 | 13 | 349 |
| Non-Hodgkin, Burkitt's & unspecified lymphoma | 459 | 26 | 4 | 11 | 21 | 521 |
| All brain and spinal | 924 | 36 | 4 | 16 | 38 | 1,018 |
| Neuroblastoma | 578 | 38 | 8 | 21 | 24 | 669 |
| Retinoblastoma | 196 | 13 | 2 | 4 | 3 | 218 |
| Wilms' tumour | 513 | 16 | 10 | 10 | 20 | 569 |
| Osteosarcoma | 130 | 7 | 1 | 3 | 9 | 150 |
| Ewing's sarcoma | 156 | 8 | 0 | 2 | 6 | 172 |
| Rhabdomyosarcoma | 406 | 10 | 4 | 14 | 14 | 448 |
| Fibrosarcoma and other soft-tissue sarcoma | 133 | 6 | 1 | 1 | 6 | 147 |
| Non-gonadal germ-cell | 84 | 4 | 1 | 2 | 7 | 98 |
| Gonadal germ-cell | 108 | 16 | 0 | 4 | 3 | 131 |
| All other malignant neoplasms | 196 | 10 | 3 | 14 | 6 | 229 |
| Total | 6,783 | 366 | 63 | 173 | 273 | 7,658 |

**Table II** Numbers (%) of registrations in selected diagnostic groups for Caucasians and Asians in three five-year age groups

| | *Caucasian* | | | |
|---|---|---|---|---|
| | *Age* | | | |
| | *0–4* | *5–9* | *10–14* | *Total* |
| ALL | 1,187(34) | 569(33) | 357(23) | 2,113(31) |
| Hodgkin's disease | 22(1) | 83(5) | 192(12) | 297(4) |
| All brain and spinal | 371(11) | 330(19) | 223(14) | 924(14) |
| Wilms' tumour | 397(11) | 102(6) | 14(1) | 513(8) |
| Rhabdomyosarcoma | 204(6) | 112(6) | 90(6) | 406(6) |
| All other malignant neoplasms | 1,303(37) | 538(31) | 689(44) | 2,530(37) |
| Total | 3,484 | 1,734 | 1,565 | 6,783 |
| | *Asian* | | | |
| | *Age* | | | |
| | *0–4* | *5–9* | *10–14* | *Total* |
| ALL | 71(37) | 32(32) | 18(25) | 121(33) |
| Hodgkin's disease | 8(4) | 10(10) | 11(15) | 29(8) |
| All brain and spinal | 12(6) | 15(15) | 9(13) | 36(10) |
| Wilms' tumour | 12(6) | 3(3) | 1(1) | 16(4) |
| Rhabdomyosarcoma | 6(3) | 1(1) | 3(4) | 10(3) |
| All other malignant neoplasms | 84(44) | 40(40) | 30(42) | 154(42) |
| Total | 193 | 101 | 72 | 366 |

**Table III** Immunophenotype of ALL among children of principal ethnic groups

| | *Caucasian* | | | | |
|---|---|---|---|---|---|
| | *Age* | | | | |
| | *0* | *1–4* | *5–9* | *10–14* | *Total* |
| Common | 12 | 668 | 312 | 157 | 1,149(54%) |
| T-cell | 4 | 66 | 76 | 81 | 227(11%) |
| B-cell | 5 | 11 | 10 | 20 | 46(2%) |
| Null | 41 | 23 | 18 | 13 | 95(4%) |
| Unknown | 24 | 333 | 153 | 86 | 596(28%) |
| Total | 86 | 1,101 | 569 | 357 | 2,113 |
| | *Asian* | | | | |
| | *Age* | | | | |
| | *0* | *1–4* | *5–9* | *10–14* | *Total* |
| Common | 2 | 41 | 18 | 5 | 66(55%) |
| T-cell | 0 | 3 | 4 | 5 | 12(10%) |
| B-cell | 0 | 1 | 0 | 1 | 2(2%) |
| Null | 4 | 1 | 3 | 1 | 9(7%) |
| Unknown | 1 | 18 | 7 | 6 | 32(26%) |
| Total | 7 | 64 | 32 | 18 | 121 |
| | *West Indian* | | | | |
| | *Age* | | | | |
| | *0* | *1–4* | *5–9* | *10–14* | *Total* |
| Common | 0 | 5 | 4 | 1 | 10(59%) |
| T-cell | 0 | 0 | 1 | 1 | 2(12%) |
| B-cell | 0 | 0 | 0 | 1 | 1(6%) |
| Null | 0 | 2 | 0 | 0 | 2(12%) |
| Unknown | 0 | 0 | 1 | 1 | 2(12%) |
| Total | 0 | 7 | 6 | 4 | 17 |

children aged 5–9, where Wilms' tumour accounted for 21% (4/19) of all cancers, compared with 5.9% in Caucasians.

Rhabdomyosarcoma was relatively infrequent among Asian children, particularly among those under 10 years of age (Tables I and II). Gonadal germ-cell tumours appeared to be more than twice as common among Asian children as among Caucasians (Table I). No substantial variations in relative frequency by ethnic group were noted for any other major diagnostic groups.

Table V shows the results of the regression analyses for diagnostic groups with a total of at least 150 cases for all ages combined. There were highly significant excesses of Hodgkin's disease among Asian children and of Wilms' tumour among West Indians. Asian children had significant deficits of Wilms' tumour and rhabdomyosarcoma. Table VI shows the corresponding results for Asian children in three 5-year age groups for selected diagnostic groups. There was a highly significant excess of Hodgkin's disease and a significant deficit of Wilms' tumour among Asians aged 0–4.

When the log linear modelling method is used for frequency data, the odds ratio is only an accurate estimate of the relative risk if the control group of all other cancers is unbiased with respect to the risk factor being analysed, which in this study is the ethnic group (Kaldor *et al.*, 1990). With this in mind, we repeated the analyses excluding from the control group all cases of Hodgkin's disease, Wilms' tumour and rhabdomyosarcoma, the three diagnostic groups which had yielded significant results. The results of the analyses were essentially unchanged.

### Discussion

In comparing patterns of cancer incidence between geographical regions or between sub-populations of the same region, the most informative statistics are incidence rates calculated from complete, population-based registries. When ascertainment of cases is incomplete or there are no reliable population data, however, comparisons of relative frequencies of tumour types between populations can yield much valuable information (Parkin *et al.*, 1988*a*).

The data from the UKCCSG register, though not population-based, represent the largest series of childhood cancer in Britain for which ethnic group has been recorded. The children in the register were all patients at specialist paediatric oncology centres, where there would be expected to be particular expertise in the diagnosis of childhood malignant disease. Histological confirmation of diagnosis was available for 95% of cases. If neuroblastoma, hepatoblastoma and germ-cell tumours diagnosed biochemically and retinoblastoma diagnosed by examination under anaesthetic are added, the proportion of confirmed diagnoses rises to 96%. The remaining 4% are predominantly intracranial tumours diagnosed radiologically. Ethnic group was recorded in all but 3.6% of cases, with little evidence of variation in the level of recording between diagnostic groups.

The patterns of incidence presented here may be compared with those found internationally, principally from the recent study coordinated by the International Agency for Research on Cancer (IARC) (Parkin *et al.*, 1988*a*).

In the IARC study, the incidence rates and relative fre-

Table IV Histological subtypes of Hodgkin's disease among Caucasian and Asian children

| | Caucasian Age 0-4 | 5-9 | 10-14 | Total | Asian Age 0-4 | 5-9 | 10-14 | Total |
|---|---|---|---|---|---|---|---|---|
| LP | 4 | 24 | 36 | 64(22%) | 1 | 1 | 3 | 5(17%) |
| MC | 9 | 25 | 40 | 74(25%) | 3 | 7 | 4 | 14(48%) |
| LD | 1 | 0 | 4 | 5(2%) | 0 | 0 | 0 | 0 |
| NS | 8 | 32 | 108 | 148(50%) | 4 | 2 | 3 | 9(31%) |
| Unknown | 0 | 2 | 4 | 6(2%) | 0 | 0 | 1 | 1(3%) |
| Total | 22 | 83 | 192 | 297 | 8 | 10 | 11 | 29 |

LP = lymphocyte predominant. MC = mixed cellularity. LD = lymphocyte depleted. NS = nodular sclerosis.

Table V Estimated relative risks by ethnic group, with Caucasians as reference group, adjusted for effects of centre, age and sex, with results of test for heterogeneity between ethnic groups

| | Ethnic group Asian | West Indian | Other and mixed | Unknown | $\chi^2$ on 4df for heterogeneity |
|---|---|---|---|---|---|
| ALL | 1.08 | 0.90 | 1.02 | 0.97 | 0.65 |
| ANLL | 0.94 | 0.51 | 1.06 | 0.92 | 1.25 |
| Hodgkin's disease | 2.09[c] | 1.85 | 0.73 | 0.96 | 12.1[a] |
| Non-Hodgkin, Burkitt's and unspecified lymphoma | 1.04 | 0.91 | 1.06 | 0.98 | 0.10 |
| All brain and spinal | 0.75 | 0.51 | 0.91 | 1.04 | 4.65 |
| Neuroblastoma | 1.19 | 1.78 | 1.31 | 1.06 | 3.58 |
| Retinoblastoma (all) | 1.23 | 1.12 | 0.80 | 0.37 | 4.8 |
| Retinoblastoma (unilateral) | 1.86 | 2.18 | 1.19 | 0.25 | 7.42 |
| Retinoblastoma (bilateral) | 0.58 | 0 | 0.41 | 0.52 | 4.50 |
| Wilms' tumour | 0.51[a] | 2.55[a] | 0.67 | 1.03 | 15.1[b] |
| Osteosarcoma | 1.10 | 0.85 | 1.31 | 1.86 | 2.60 |
| Ewing's sarcoma | 1.00 | 0 | 0.55 | 0.86 | 4.65 |
| Rhabdomyosarcoma | 0.44[a] | 0.84 | 1.25 | 0.71 | 10.2[a] |

Note: Estimates for retinoblastoma not adjusted for centre or age. [a]$P<0.05$. [b]$P<0.01$. [c]$P<0.001$.

Table VI Estimated age-specific relative risks for Asian children, with Caucasians as reference group, adjusted for effects of centre and sex

| | Age at diagnosis (years) 0-4 | 5-9 | 10-14 |
|---|---|---|---|
| ALL | 1.11 | 0.99 | 1.13 |
| Hodgkin's disease | 6.67[b] | 2.18 | 1.25 |
| All brain and spinal | 0.63 | 0.82 | 0.88 |
| Wilms' tumour | 0.49[a] | 0.47 | 1.53 |
| Rhabdomyosarcoma | 0.50 | 0.15 | 0.75 |

[a]$P<0.05$. [b]$P<0.001$.

quency of childhood ALL in India, Pakistan and Bangladesh were lower than in Western countries. The marked peak of incidence among children aged 1-4 which is characteristic of Caucasian populations were also absent (Parkin et al., 1988b). By contrast, the Asian children in the present study had a similar relative frequency of ALL to Caucasian children which, on the assumption of similar incidence for all cancers combined, translates into a similar incidence rate. The age distributions of ALL among children of the two ethnic groups were also very similar, the Asians exhibiting the same predominance of patients aged under five as Caucasians. These results confirm the suggestions of two much smaller, earlier studies that Asian children in Britain experience a pattern of incidence of ALL similar to that of Caucasian children (Oakhill & Mann, 1983; Hill et al., 1984). The relatively high incidence of ALL among young children in Western populations is accounted for largely by the pronounced peak in the incidence of the common-ALL phenotype at this age (Greaves, 1984). In the present series, Asian children had a similar proportion of common-ALL to Caucasians, further evidence for a similar pattern of incidence in the two ethnic groups which in turn suggests similar risk factors for this disease. The relative frequency of ALL among West Indian children was slightly lower than among Caucasians, with less evidence of a peak incidence early in life. In the United States the incidence of ALL in Blacks is only half that in Whites and the age peak is less pronounced, while African registries in the IARC study had the lowest incidence of ALL (Parkin et al., 1988b). The less marked difference in patterns of occurrence of ALL between Caucasian and Black children in Britain compared with the United States may reflect a smaller difference in socioeconomic status between the ethnic groups but the number of West Indian children in this study is much smaller than the number of Black children in American series.

The incidence of childhood Hodgkin's disease appears to be highest in western Asia, extending as far east as Pakistan and north-west India, and substantial numbers of cases occur elsewhere in India and Bangladesh (Stiller & Parkin, 1990a). In this region, as in most developing countries, the steep rise in incidence during early adolescence which is found in Western populations does not occur, and mixed cellularity is the predominant histological subtype. The data relating to Hodgkin's disease among Asian children in the present study, with a high relative frequency overall, an especially high relative risk compared to Caucasians in the youngest age group and a predominance of mixed cellularity disease, suggest that the incidence rates and patterns of occurrence of childhood Hodgkin's disease in Asian children are very similar in Britain and in the Indian sub-continent. This persistence of a distinct pattern of occurrence of Hodgkin's disease among children of a particular ethnic group in different countries might suggest ethnically determined genetic variations in predisposition to this tumour. The high incidence among children in developing countries has been interpreted in terms of an infectious aetiology related to poor living conditions (Gutensohn & Cole, 1977) and interracial variations in Hodgkin's disease in developed countries could also be accounted for by ethnic differences in socio-economic status (Glaser, 1991).

In the present study neuroblastoma appeared to be slightly more common among Asian and West Indian children than among Caucasians, though the excesses were not statistically significant. In the West Midlands during 1957–86, however, there was an apparent deficit of neuroblastoma among Afro-Caribbean children (Muir *et al.*, 1990). The incidence of neuroblastoma was slightly lower among Blacks than Whites in the United States but the rates for American Blacks were similar to those found in many European countries. Data from African registries were difficult to interpret but were consistent with a somewhat lower incidence rate. The incidence of neuroblastoma in Bombay was also low.

Some of the highest incidence rates for retinoblastoma in the IARC study occurred among Blacks in Africa and the United States, and also in Bombay (Parkin *et al.*, 1988*b*). There was also a high relative frequency of retinoblastoma in several African and Asian series for which incidence rates could not be calculated. Where information on laterality was available in high-incidence areas, the increased rate was apparently accounted for by an excess of unilateral (predominantly sporadic) cases rather than of bilateral (hereditary) tumours. The present series only contained two West Indian children with retinoblastoma, both of whom had unilateral tumours. The excess of Asian children was entirely due to unilateral retinoblastoma, repeating the pattern found in Asian registries.

At one time Wilms' tumour was thought to be an 'index tumour of childhood', with approximately constant incidence in all populations (Innis, 1972). The present data provide further evidence of substantial variations in the occurrence of Wilms' tumour. The highest incidence has been found in Black populations both in Africa and in the United States (Parkin *et al.*, 1988*b*; Stiller & Parkin, 1990*b*). Blacks with Wilms' tumour have been reported as being slightly older than Whites in the United States (Breslow *et al.*, 1988) though there was little evidence of differences in the age-distribution between Blacks and Whites in the IARC study. Wilms' tumour is relatively rare in the Indian sub-continent (Stiller & Parkin, 1990*b*). In the present series, West Indians had a substantially higher relative frequency of Wilms' tumour which was most marked among older children. Asian children, on the other hand, had a low relative frequency compared with Caucasians. Thus it seems likely that, for children of south Asian ethnic origin as well as for Blacks, the incidence of Wilms' tumour is unaffected by migration, a pattern also observed for children of Far Eastern origin in east Asia and the United States (Stiller & Parkin, 1990*b*).

The absence of Ewing's sarcoma among West Indian children is consistent with the very low incidence of this tumour among Blacks in Africa and the United States (Parkin *et al.*, 1988*b*), though fewer than two cases would have been expected if West Indian children had similar incidence to Caucasians.

Few patterns of incidence of childhood soft tissue sarcoma emerged in the IARC study, though the incidence of rhabdomyosarcoma appeared to be lower in south Asia than in populations of European origin (Parkin *et al.*, 1988*b*). It is impossible to be sure how closely the pattern of incidence among Asians in Britain, with a marked deficit of rhabdomyosarcoma among younger children, parallels that in the Indian sub-continent, since most of the south Asian series included a relatively large number of soft-tissue sarcomas of unspecified type (Parkin *et al.*, 1988*a*).

Gonadal tumours are apparently more common in east Asia than in most other regions (Parkin *et al.*, 1988*b*) but their incidence elsewhere in Asia was unremarkable. The excess of gonadal germ-cell tumours among Asian children in the present study is therefore difficult to interpret, particularly since the total number of cases was too small to include in the regression analyses.

Overall, two distinct patterns of incidence are found in the data presented here. The first pattern occurs in relation to ALL, for which the incidence appears to vary little between ethnic groups within the British Isles, but the incidence among Asians in the Indian sub-continent and Blacks in Africa shows a markedly different pattern from that observed in Britain. These results suggest that the incidence of ALL depends primarily on environmental factors associated with geographical location, lending weight to two hypotheses concerning its aetiology. The first hypothesis is that of Ramot and Magrath (1982) who proposed that patterns of lymphoid malignancy are environmentally determined, particularly relating to socio-economic factors. The second is that of Greaves (1988), under which patterns of infection in early life are linked to the development of common ALL.

The second pattern of incidence appears to apply to Hodgkin's disease, retinoblastoma and Wilms' tumour. For these diagnostic groups, the incidence appears to differ primarily between ethnic groups, and to remain substantially unchanged by migration. Not only retinoblastoma and Wilms' tumour but also Hodgkin's disease in early childhood thus appear likely to have a strong genetic component to their aetiology. If environmental factors are involved, they may be associated more with aspects of the way of life which are distinctive for particular ethnic groups, rather than with specific regions of the world.

We thank the many members of the UKCCSG who provided the information on which this paper is based. We are grateful to Mrs E.M. Roberts for secretarial help. The Childhood Cancer Research Group is supported by the Department of Health and the Scottish Home and Health Department. The UKCCSG is supported by the Cancer Research Campaign.

### References

BIRCH, J.M. & MARSDEN, H.B. (1987). A classification scheme for childhood cancer. *Int. J. Cancer*, **40**, 620.

BRESLOW, N.E., BECKWITH, J.B., CIOL, M. & SHARPLES, K. (1988). Age distribution of Wilms' Tumor: Report from the National Wilms' Tumor Study. *Cancer Res.*, **48**, 1653.

BRESLOW, N.E. & DAY, N.E. (1987). Statistical methods in cancer research. Volume II, the design and analysis of cohort studies. *IARC Scientific Publications, No. 82*, Lyon, IARC.

GLASER, S.L. (1991). Black-white differences in Hodgkin's disease incidence in the United States by age, sex, histology subtype and time. *Int. J. Epidemiol.*, **20**, 68.

GOODMAN, M.T., YOSHIZAWA, C.N. & KOLONEL, L.N. (1989*a*). Incidence trends and ethnic patterns for childhood leukaemia in Hawaii: 1960–1984. *Br. J. Cancer*, **60**, 93.

GOODMAN, M.T., YOSHIZAWA, C.N. & KOLONEL, L.N. (1989*b*). Ethnic patterns of childhood cancer in Hawaii between 1960 and 1984. *Cancer*, **64**, 1758

GREAVES, M.F. (1984). Subtypes of acute lymphoblastic leukaemia: implications for the pathogenesis and epidemiology of leukaemia. In *Pathogenesis of Leukemias and Lymphomas: Environmental Influences*, Magrath, I.T., O'Conor, G.T. & Ramot, B. (eds), p. 129, Raven Press: New York.

GREAVES, M.F. (1988). Speculations on the cause of childhood acute lymphoblastic leukemia. *Leukemia*, **2**, 120.

GUTENSOHN, N. & COLE, P. (1977). Epidemiology of Hodgkin's disease in the young. *Int. J. Cancer*, **19**, 595.

HILL, F.G.H., AL RUBEI, K. & TRUMPER, P.A. (1984). An epidemiological study of childhood acute lymphoblastic leukaemia in the West Midlands of the United Kingdom, showing seasonal variation in T-cell disease. In *Pathogenesis of Leukemias and Lymphomas: Environmental Influences*, Magrath, I.T., O'Conor, G.T. & Ramot, B. (eds), p. 147, Raven Press: New York.

INNIS, M.D. (1972). Nephroblastoma: possible index cancer of childhood. *Med J. Aust.*, **1**, 18.

KALDOR, J., KHLAT, M., PARKIN, D.M., SHIBOSKI, S. & STEINITZ, R. (1990). Log-linear models for cancer risk among migrants. *Int. J. Epidemiol.*, **19**, 233.

KRAMER, S., MEADOWS, A.T. & JARRETT, P. (1983). Incidence of childhood cancer: experience of a decade in a population-based cancer registry. *J. Natl Cancer Inst.*, **70**, 49.

MUIR, K.R., HUDDART, S.N., BARRANTES, J., PARKES, S.E. & MANN, J.R. (1990). Relative occurrence of neuroblastoma and Wilms' tumour in ethnic subgroups in the West Midlands Health Authority Region. *Arch. Dis. Child.*, **65**, 1380.

OAKHILL, A. & MANN, J.R. (1983). Poor prognosis of acute lymphoblastic leukaemia in Asian children living in the United Kingdom. *Br. Med. J.*, **286**, 839.

PARKIN, D.M., STILLER, C.A., BIEBER, A., DRAPER, G.J., TERRACINI, B. & YOUNG, J.L. (eds) (1988*a*). International Incidence of Childhood Cancer. *IARC Scientific Publications No. 87*, IARC: Lyon.

PARKIN, D.M., STILLER, C.A., DRAPER, G.J. & BIEBER, C.A. (1988*b*). The international incidence of childhood cancer. *Int. J. Cancer*, **42**, 511.

PAYNE, C.D. (ed.) (1987). *The generalised linear interactive modelling (GLIM) system.* Release 3.77. Numerical Algorithms Group: Oxford.

RAMOT, B. & MAGRATH, I. (1982). Hypothesis: The environment is a major determinant of the immunological subtype of lymphoma and acute lymphoblastic leukaemia in children. *Br. J. Haematol.*, **52**, 183.

SANDERS, B.M., DRAPER, G.J. & KINGSTON, J.E. (1988). Retinoblastoma in Great Britain, 1969–80: incidence, treatment and survival. *Br. J. Ophthalmol.*, **72**, 576.

STILLER, C.A. (1988). Centralisation of treatment and survival rates for cancer. *Arch. Dis. Child.*, **63**, 23.

STILLER, C.A. & PARKIN, D.M. (1990*a*). International variations in the incidence of childhood lymphomas. *Paediatr. Perinatal Epidemiol.*, **4**, 302.

STILLER, C.A. & PARKIN, D.M. (1990*b*). International variations in the incidence of childhood renal tumours. *Br. J. Cancer*, **62**, 1026.

YOUNG, J.L., GLOECKLER RIES, L., SILVERBERG, E., HORM, J.A. & MILLER, R.W. (1986). Cancer incidence, survival and mortality for children younger than age 15 years. *Cancer*, **58**, 598.


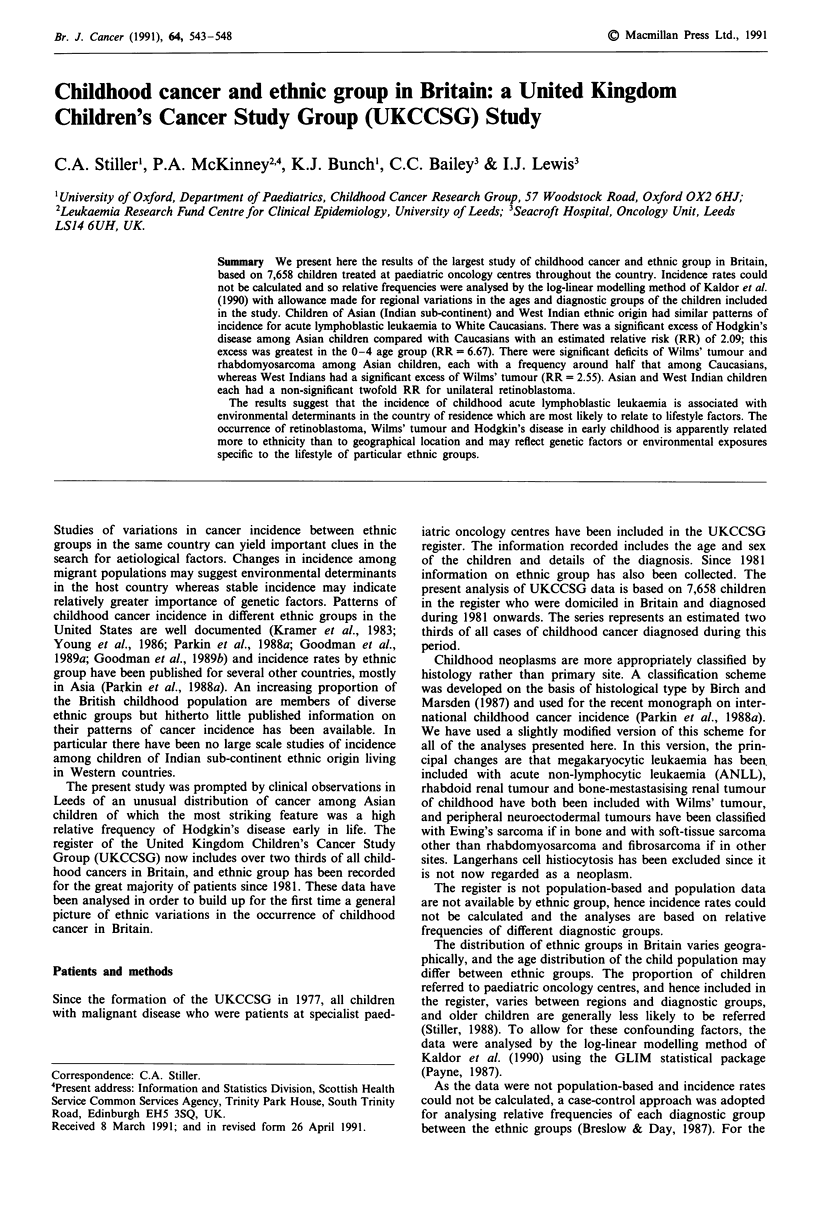

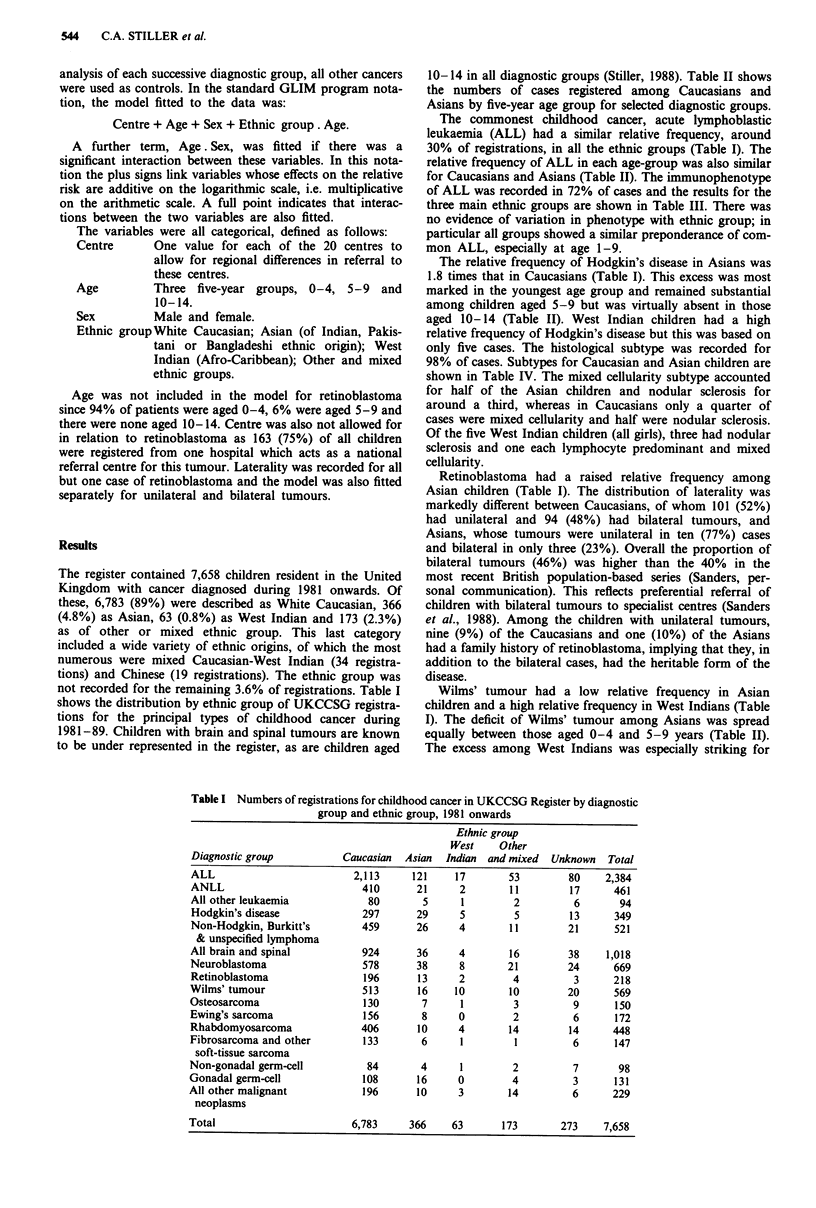

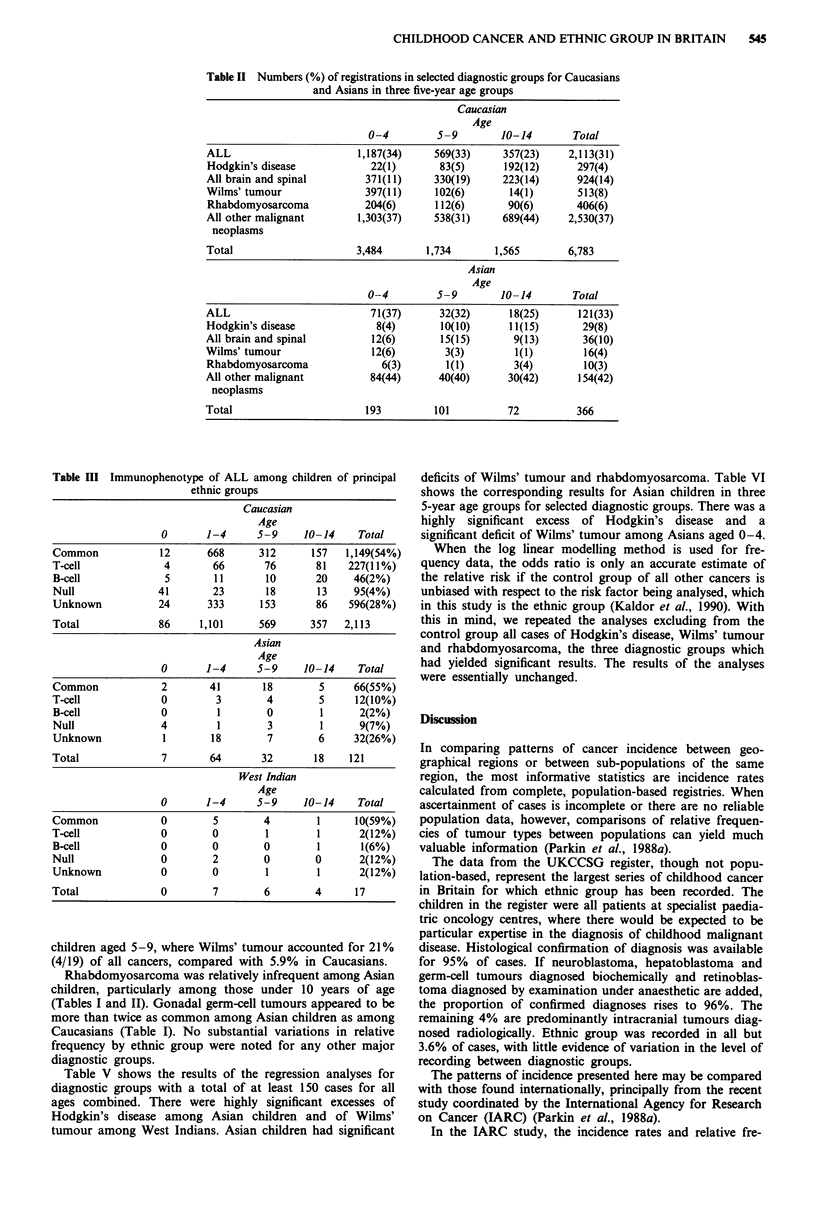

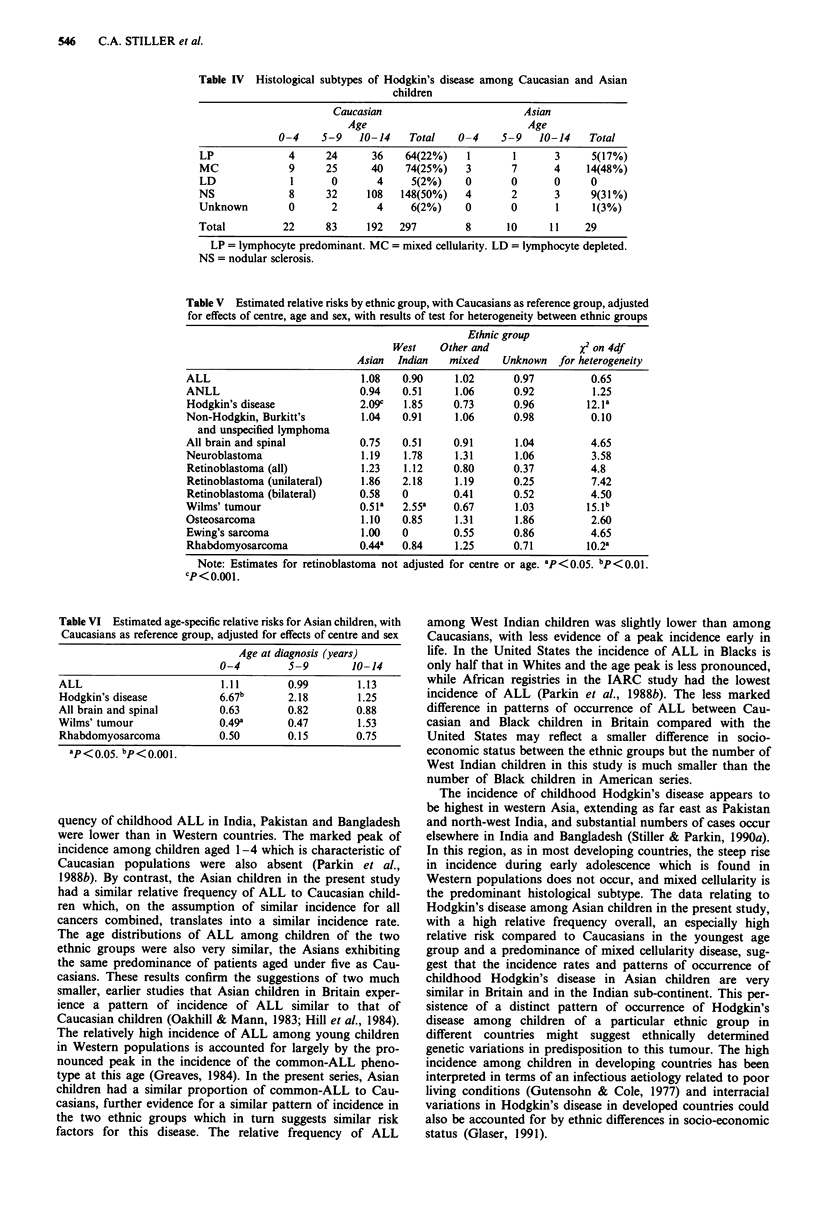

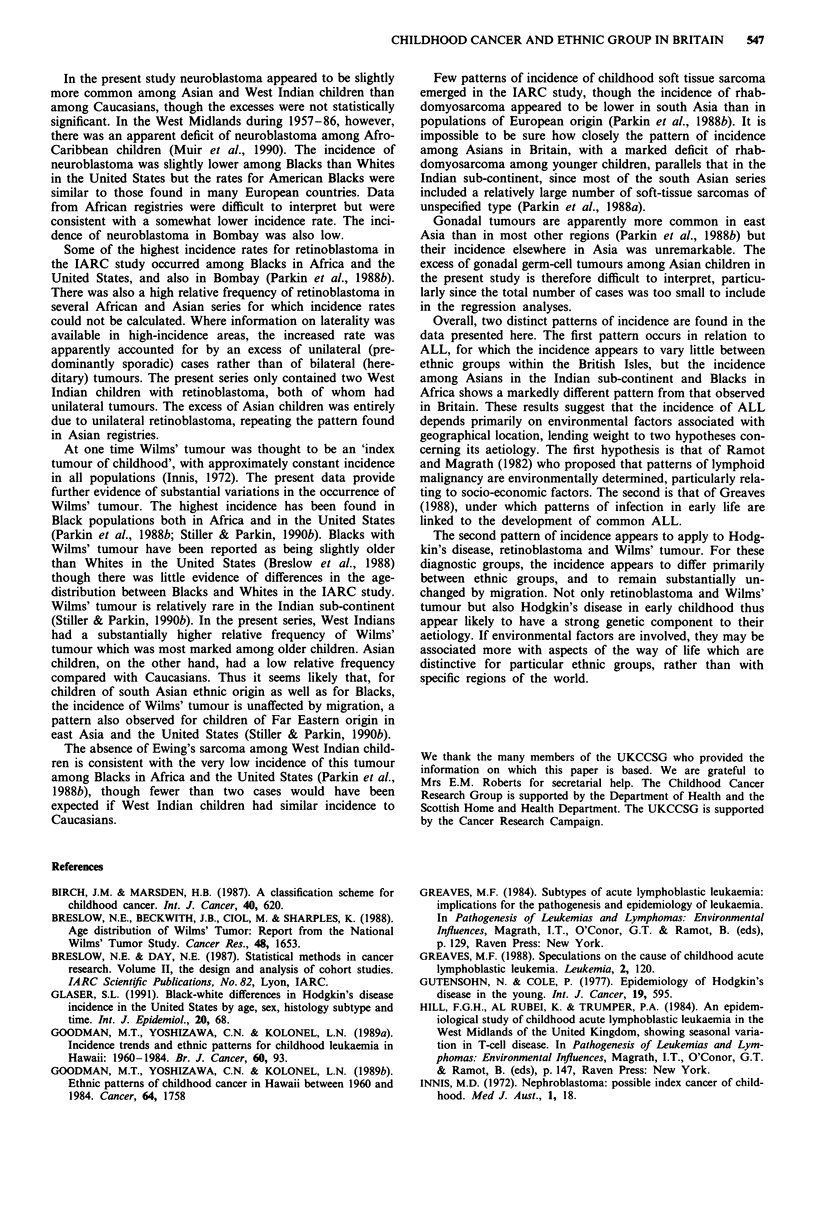

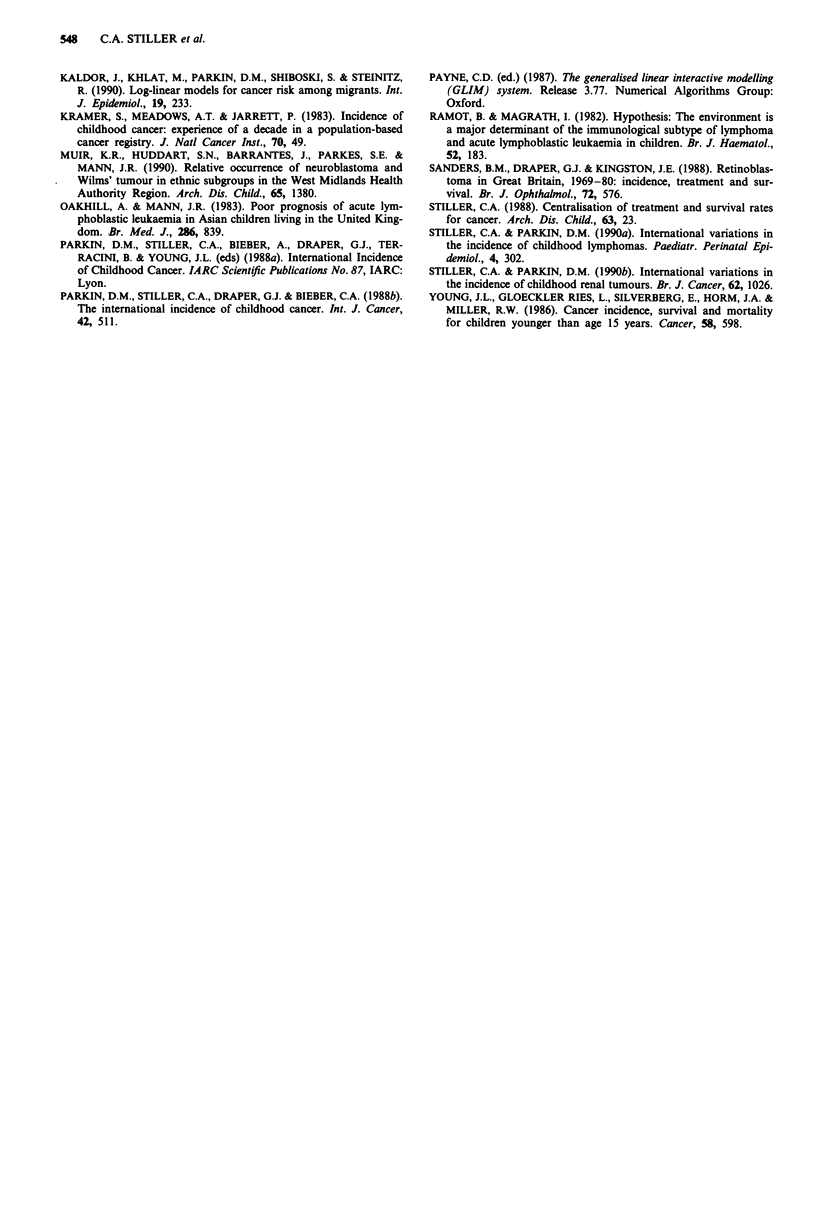


## References

[OCR_00671] Birch J. M., Marsden H. B. (1987). A classification scheme for childhood cancer.. Int J Cancer.

[OCR_00675] Breslow N., Beckwith J. B., Ciol M., Sharples K. (1988). Age distribution of Wilms' tumor: report from the National Wilms' Tumor Study.. Cancer Res.

[OCR_00685] Glaser S. L. (1991). Black-white differences in Hodgkin's disease incidence in the United States by age, sex, histology subtype and time.. Int J Epidemiol.

[OCR_00695] Goodman M. T., Yoshizawa C. N., Kolonel L. N. (1989). Ethnic patterns of childhood cancer in Hawaii between 1960 and 1984.. Cancer.

[OCR_00690] Goodman M. T., Yoshizawa C. N., Kolonel L. N. (1989). Incidence trends and ethnic patterns for childhood leukaemia in Hawaii: 1960-1984.. Br J Cancer.

[OCR_00707] Greaves M. F. (1988). Speculations on the cause of childhood acute lymphoblastic leukemia.. Leukemia.

[OCR_00711] Gutensohn N., Cole P. (1977). Epidemiology of hodgkin's disease in the young.. Int J Cancer.

[OCR_00723] Innis M. D. (1972). Nephroblastoma: possible index cancer of childhood.. Med J Aust.

[OCR_00729] Kaldor J., Khlat M., Parkin D. M., Shiboski S., Steinitz R. (1990). Log-linear models for cancer risk among migrants.. Int J Epidemiol.

[OCR_00734] Kramer S., Meadows A. T., Jarrett P., Evans A. E. (1983). Incidence of childhood cancer: experience of a decade in a population-based registry.. J Natl Cancer Inst.

[OCR_00739] Muir K. R., Huddart S. N., Barrantes J., Parkes S. E., Mann J. R. (1990). Relative occurrence of neuroblastoma and Wilms' tumour in ethnic subgroups in the West Midlands Health Authority Region.. Arch Dis Child.

[OCR_00745] Oakhill A., Mann J. R. (1983). Poor prognosis of acute lymphoblastic leukaemia in Asian children living in the United Kingdom.. Br Med J (Clin Res Ed).

[OCR_00756] Parkin D. M., Stiller C. A., Draper G. J., Bieber C. A. (1988). The international incidence of childhood cancer.. Int J Cancer.

[OCR_00766] Ramot B., Magrath I. (1982). Hypothesis: the environment is a major determinant of the immunological sub-type of lymphoma and acute lymphoblastic leukaemia in children.. Br J Haematol.

[OCR_00772] Sanders B. M., Draper G. J., Kingston J. E. (1988). Retinoblastoma in Great Britain 1969-80: incidence, treatment, and survival.. Br J Ophthalmol.

[OCR_00777] Stiller C. A. (1988). Centralisation of treatment and survival rates for cancer.. Arch Dis Child.

[OCR_00786] Stiller C. A., Parkin D. M. (1990). International variations in the incidence of childhood renal tumours.. Br J Cancer.

[OCR_00791] Young J. L., Ries L. G., Silverberg E., Horm J. W., Miller R. W. (1986). Cancer incidence, survival, and mortality for children younger than age 15 years.. Cancer.

